# VAE deep learning model with domain adaptation, transfer learning and harmonization for diagnostic classification from multi-site neuroimaging data

**DOI:** 10.3389/fninf.2025.1553035

**Published:** 2025-09-11

**Authors:** Gopikrishna Deshpande, Bonian Lu, Nguyen Huynh, D. Rangaprakash

**Affiliations:** ^1^Department of Electrical and Computer Engineering, Auburn University Neuroimaging Center, Auburn University, Auburn, AL, United States; ^2^Department of Psychological Sciences, Auburn University, Auburn, AL, United States; ^3^Alabama Advanced Imaging Consortium, Birmingham, AL, United States; ^4^Center for Neuroscience, Auburn University, Auburn, AL, United States; ^5^Department of Heritage Science and Technology, Indian Institute of Technology, Hyderabad, India; ^6^Department of Psychiatry, National Institute of Mental Health and Neurosciences, Bangalore, India; ^7^Athinoula A. Martinos Center for Biomedical Imaging, Massachusetts General Hospital, Harvard Medical School, Boston, MA, United States

**Keywords:** functional connectivity, Autism Spectrum Disorders, domain adaptation, variational autoencoder, machine learning prediction

## Abstract

In large public multi-site fMRI datasets, the sample characteristics, data acquisition methods, and MRI scanner models vary across sites and datasets. This non-neural variability obscures neural differences between groups and leads to poor machine learning based diagnostic classification of neurodevelopmental conditions. This could be potentially addressed by domain adaptation, which aims to improve classification performance in a given target domain by utilizing the knowledge learned from a different source domain by making data distributions of the two domains as similar as possible. In order to demonstrate the utility of domain adaptation for multi-site fMRI data, this research developed a variational autoencoder—maximum mean discrepancy (VAE-MMD) deep learning model for three-way diagnostic classification: (i) Autism, (ii) Asperger's syndrome, and (iii) typically developing controls. This study chooses ABIDE-II (Autism Brain Imaging Data Exchange) dataset as the target domain and ABIDE-I as the source domain. The results show that domain adaptation from ABIDE-I to ABIDE-II provides superior test accuracy of ABIDE-II compared to just using ABIDE-II for classification. Further, augmenting the source domain with additional healthy control subjects from Healthy Brain Network (HBN) and Amsterdam Open MRI Collection (AOMIC) datasets enables transfer learning and improves ABIDE-II classification performance. Finally, a comparison with statistical data harmonization techniques, such as ComBat, reveals that domain adaptation using VAE-MMD achieves comparable performance, and incorporating transfer learning (TL) with additional healthy control data substantially improves classification accuracy beyond that achieved by statistical methods (such as ComBat) alone. The dataset and the model used in this study are publicly available. The neuroimaging community can explore the possibility of further improving the model by utilizing the ever-increasing amount of healthy control fMRI data in the public domain.

## 1 Introduction

Neuroimaging has been widely used to identify structural and functional alterations in the cerebral cortex and disrupted functional connectivity in ASD (DeRamus et al., 2014; Dichter, 2012; Holmes et al., 2015; Horwitz et al., 1988; Koshino et al., 2008; Minshew and Keller, 2010; Pantelis et al., 2015; Rakić et al., 2020; Verly et al., 2014; Washington et al., 2020; Xu et al., 2019). More recently, machine learning models have been applied to neuroimaging data for diagnostic classification. Deep learning models outperform traditional machine learning methods in identifying individuals with neurodevelopmental conditions, including autism (Li et al., 2018; Di Martino et al., 2017; Cao et al., 2021; Duc et al., 2020; Eslami et al., 2019; Panta et al., 2016; Plis et al., 2014; Subah et al., 2021). However, deep learning models require larger sample sizes to avoid overfitting (Karimi et al., 2020). Large public databases, such as ABIDE (Autism Brain Imaging Data Exchange), have aided deep learning models in this endeavor. However, such large public databases have been assembled *post-hoc* and contain different sources of non-neural variabilities, such as various sites using different scanners and protocols. Typically, the samples from different scanners or acquisition protocols do not follow the same distribution in most cases (Nielsen et al., 2013). Moreover, if the test data and training data are drawn from different independent distributions, the performance of deep-learning, as well as traditional machine learning models, will be degraded (Li et al., 2018; Lanka et al., 2020). This study proposes a domain adaptation technique to improve the classification performance in a target domain to address this issue. The proposed method utilizes the knowledge learned from the source domain and makes the data distributions in source and target domains as similar as possible (Ben-David et al., 2010; Li et al., 2020; Zhou et al., 2018).

**Figure 1:**
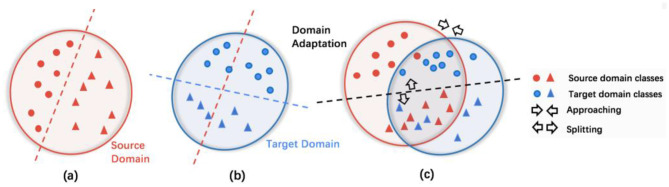
**(a, b)** Are the classifiers before domain adaptation in the source and target domains, respectively. The domain adaptation process aims to reduce the domain divergence by maximizing the domain confusion as well as minimizing the classification loss in **(c)**. The red line in **(a)** illustrates the decision boundary from training a source domain classifier. When this is transferred to the target domain as is, it is sub-optimal. The blue line in **(b)** illustrates the desired decision boundary from training a target domain classifier. The black line in **(c)** shows the decision boundary result from training a domain adaptation classifier.

To understand domain adaptation, as illustrated in Figure 1, the data distributions differ in the source and the target domains, although the two groups are separable in both domains that are taken independently. However, the classifier learned from the source domain (the red dotted line in Figure 1a) cannot directly be transferred to the target domain (Figure 1b). This affects the generalizability of the classifier. Thus, the objective of domain adaptation is to learn the differences in data distributions and improve the target domain classifier (black dotted line in Figure 1c) by jointly optimizing the classification and domain fusion (illustrated by approaching and splitting arrows in Figure 1c; Tzeng et al., 2017). In neuroimaging research, the transductive scenario assumes that the dataset from the source domain has annotated labels from an expert, and the dataset from the target domain may not have labels. The domain adaptation approach is jointly optimized to minimize the domain-shift effect across source domain data and target domain data (Kushibar et al., 2021).

Multiple studies (Gholami et al., 2020; Hoffman et al., 2018; Zhao et al., 2019; Bickel and Brückner, 2007; Rahimi and Recht, 2008; Simonyan and Zisserman, 2015) proposed different frameworks to exploit commonalities between different data domains to achieve domain adaptation in various areas (Csurka, 2017; Tzeng et al., 2017; Ghafoorian et al., 2017; Ganin et al., 2016). However, a limited number of end-to-end deep learning models incorporating domain adaptation have been developed for neuroimaging data (Hangya et al., 2018; Ilse et al., 2020; Purushotham et al., 2017; Wachinger et al., 2016). For example, Li et al. (2020) proposed a domain adaptation framework for federated datasets across different sites of the ABIDE dataset. Similarly, another study (Zhou et al., 2018) formulated the DawfMRI framework, which revealed additional insights into psychological similarity among the OpenfMRI project databases. Both studies aligned different data domains into one common embedding space followed by biomarker identification. But it was achieved by training each local model individually and integrating them with an ensemble strategy. Since this is not implemented as a single deep learning model; therefore, the complexity of a model increases, and the ease of use decreases. Thus, training and optimizing a deep learning model becomes more challenging.

Existing domain adaptation approaches applied in neuroimaging-based diagnostic classification primarily employ supervised learning techniques. For example, a previous study (Cheng et al., 2012) used the labeled Alzheimer's Disease Neuroimaging Initiative (ADNI) database to propose a robust domain transfer support vector machine (DTSVM) model to classify mild cognitive impairment (MCI). Another study (Khan et al., 2019) utilized the supervised domain adaptation (SDA) method on the pre-trained VGG network and used labeled MRI data to fine-tune the Alzheimer's disease prediction model. Nevertheless, developing prediction models on medical data is marred by the complex labeling process that is not always accurate (Litjens et al., 2017). This is because the diagnosis of psychiatric disorders is based on behavior and not objective biomarkers that can make the labels less accurate for marginal cases and the stratification of individuals in spectrum disorders. Therefore, unsupervised domain adaptation (UDA) has recently gained importance because label scarcity is a common challenge across medical imaging studies (Choudhary et al., 2020).

UDA techniques have been used to address potential inaccuracies in labels (Karimi et al., 2020; Haeusser et al., 2017; Kamnitsas et al., 2017; Mahmood et al., 2018) and to increase the statistical power of analysis by adding more unlabeled data (Guan and Liu, 2021). Combining supervised and unsupervised learning domain adaptation methods has improved discriminative prediction accuracy (Choudhary et al., 2020). This method requires limited labeled data or no labeled data from the target domain (Madani et al., 2018). Moreover, Semi-supervised domain adaptation methods have been proposed and tested on deep learning benchmark data (Belhaj et al., 2018; Chen and Chien, 2015). Variational auto-encoder (VAE; Kingma et al., 2014) outperformed all the other semi-supervised domain adaptation methods. VAE model is robust against high-dimensional input data and can learn various distributions flexibly. Another study (Louizos et al., 2016) used the learning features of VAE to develop a variational fair autoencoder (VFA). Moreover, VFA was proposed to learn the features that are invariant to noisy nuisance factors but retain useful information as much as possible. However, previous literature on the semi-supervised learning approach in neurodevelopmental condition classification is scarce. Therefore, this study used unlabeled data during training and a semi-supervised approach to achieve domain adaptation in the target domain.

This study proposed to use variational and adversarial classification frameworks for domain adaptation by training labeled data in the source domain and unlabeled data in the target domain. A variational inference model was used to learn the invariant representations across information from different sites of the ABIDE dataset while retaining the discriminative information in the classification task. This research applied a model based on VAE to separate latent feature representations and domain variables. However, some dependencies can remain if the labels of data points are correlated with the domain variable, which can “leak” some of the domain information into the latent feature representation, resulting in dependency. Thus, the proposed model uses a “maximum mean discrepancy” (Gretton et al., 2012) regularization term to penalize the distances between the latent probability distribution across the source and target domains. A maximum mean discrepancy is a measurement of divergence between two distributions. During the adversarial training procedure, the domain “confusion” is maximized to ensure that the features are domain invariant, and the classification of Autism Spectrum Disorder (ASD) is also optimized.

Moreover, to augment domain adaptation and improve the generalizability of the classifier, we included more data in the source domain from two datasets: (i) the Healthy Brain Network (HBN^1^; Alexander et al., 2017), and (ii) the Amsterdam Open MRI Collection (AOMIC^2^; Snoek et al., 2021). HBN provides the research community with a large-scale dataset of over 10,000 healthy children and adolescents (ages 5–21) and shares the dataset through an open data-sharing mode. Only a small subset of the subjects had an MRI from New York City area. This dataset was acquired to detect and characterize pathologic processes in the developing human brain (Alexander et al., 2017). HBN data were collected from three sites: (i) Citigroup Biomedical Imaging Center (CBIC), (ii) Staten Island (SI), and (iii) Rutgers University (RU).

AOMIC, on the other hand, contains large-scale resting-state fMRI data from healthy individuals collected at the University of Amsterdam over the past decade. AOMIC publicly provided both raw and well-established pre-processed forms of three datasets: (i) PIOP1 (Population Imaging of Psychology), (ii) PIOP2, and (iii) ID1000. Each of them has specific data acquisition protocols and participants. From the demographic information in Table 1, the age range of HBN and AOMIC are close to ABIDE I and ABIDE II. We included these two databases in the domain adaptation model to increase the variety of data distribution and enhance the model's generalizability.

**Table 1:** ABIDE I data pooled from 15 different sites (and 18 cohorts, since some sites had more than one cohort), and ABIDE II from 11 sites.

**Database**	**Acquisition site**	**Subjects**	**Age mean**	**Age std**.	**Male**	**Female**
ABIDE I	CALTECH	32	26.79	9.6	25	7
	CMU	27	26.59	5.58	21	6
	KKI	55	10.1	1.31	42	13
	LEUVEN_1	29	22.59	3.49	29	0
	LEUVEN_2	35	14.16	1.4	27	8
	Max	57	26.16	11.98	50	7
	NYU	179	15.39	6.59	142	37
	OLIN	36	16.81	3.44	31	5
	PITT	57	18.9	6.82	49	8
	SBL	24	33	6.7	24	0
	SDSU	32	14.35	1.85	25	7
	TRINITY	42	16.84	3.63	42	0
	UCLA_1	73	13.16	2.38	63	10
	UCLA_2	26	12.49	1.5	24	2
	UM_1	107	13.43	2.87	83	24
	UM_2	35	15.96	3.27	33	2
	USM	100	22.14	7.67	100	0
	YALE	42	12.96	2.8	30	12
**Total**	**988**	**18.43**	**7.82**	**840**	**148**
ABIDE II	GU	104	10.68	1.62	69	35
	KKI	197	10.34	1.27	128	69
	NYU	27	6.78	1.07	24	3
	OHSU	91	10.88	1.99	56	35
	ONRC	43	23.33	3.85	31	12
	SDSU	23	13.91	3.85	20	3
	TCD	19	14.45	2.67	19	0
	UCD	32	14.78	1.83	24	8
	UCLA	32	10.7	2.36	26	6
	USM	32	21.37	7.74	27	5
	UMIA	23	9.8	2.02	17	6
	**Total**	**623**	**13.37**	**2.75**	**441**	**182**
AOMIC	PIOP1	216	21.96	1.91	29	44
	PIOP2	226	21.96	1.79	96	129
	**Total**	**442**	**21.96**	**1.85**	**125**	**173**
HBN	CBIC	287	10.75	3.73	188	99
	SI	345	11.13	3.82	195	150
	RU	753	9.92	0.42	501	252
	**Total**	**1,385**	**10.60**	**2.66**	**884**	**501**

The acquisition sites include: CALTECH, California Institute of Technology; CMU, Carnegie Mellon University; KKI, Kennedy Krieger Institute; LEUVEN, University of Leuven; MAX, Ludwig Maximilians University Munich; NYU, NYU Langone Medical Center; OLIN, Olin Institute of Living at Hartford Hospital; PITT, University of Pittsburgh School of Medicine; SBL, Social Brain Lab BCN NIC UMC Groningen and Netherlands Institute for Neurosciences; SDSU, San Diego State University; TRINITY, Trinity Center for Health Sciences; UCLA, University of California, Los Angles; UM, University of Michigan; USM, University of Utah School of Medicine; YALE, Yale Child Study Center; GU, Georgetown University; OHSU, Oregon Health and Science University; ONRC, Olin Neuropsychiatry Research Center; TCD, Trinity Center for Health Sciences; UCD, University of California Davis; UM, University of Miami. Across different acquired sites, the age and gender distributions change considerably. Both AOMIC and HBN data have had multiple releases.

We compare and contrast the proposed method with ComBat harmonization (Johnson et al., 2007), which is a statistical technique used to reduce the divergence of data distributions from multi-site MRI data. This is considered a current gold standard; therefore, we compared and combined the ComBat harmonization method with the proposed deep learning approach (Johnson et al., 2007). ComBat harmonization has been applied to neural imaging data across scanners and focuses on dealing with the variability of parameters' distributions to pool them together sites (Fortin et al., 2018). ComBat was also proposed to correct for site effects in functional measurements from multi-site fMRI data (Yu et al., 2018). Therefore, we applied ComBat harmonization to the input data as one of the methods to reduce the domain shift.

Moreover, identifying important imaging features or diagnostic classification is crucial for ASD biomarker discovery and diagnosis (Zhao et al., 2020; Traut et al., 2022). The interpretation of the correlation between domain adaptation and selected features is challenging (Gu et al., 2011; Li et al., 2016; Schölkopf et al., 2007; Sun et al., 2019). In this study, imaging features in the VAE-based model are difficult to trace back from the output layer to the input layer because of the continuous Gaussian latent variables in the latent space (Li et al., 2009). Thus, we propose a statistical method to identify such imaging features.

Based on the information presented above, we summarize four major aspects of the proposed framework:

1. We use a VAE-MMD model for domain adaptation in multi-site fMRI data for predicting the diagnostic labels from fMRI functional connectivity (FC) data. We demonstrate that domain adaptation from the first release of the ABIDE dataset (ABIDE I) to the second release (ABIDE II) will improve ABIDE II's classification performance compared to performing classification solely on ABIDE II.
2. We compare and contrast statistical (ComBat) with deep learning (VAE-MMD) approaches for domain adaptation.
3. We test whether additional data in the source domain, specifically healthy control data, will augment domain adaptation and improve the generalizability of the classifier in achieving better accuracy in the target domain of ABIDE II. Given a large amount of healthy control data available in the public domain, this transfer learning approach could potentially be used to substantially improve diagnostic classification in relatively smaller public datasets obtained from individuals with neurodevelopmental conditions, such as ASD.
4. We extract and identify imaging features diagnostically important for ASD prediction across different fMRI data distributions.

## 2 Methods

### 2.1 The fundamental algorithm of a neural network

#### 2.1.1 Multi-layer perceptron (MLP)

Deep learning algorithms have complex mathematical structures with several processing layers that can extract data features into various abstraction layers. The building block of a deep neural network (DNN) and a multi-layer perceptron (MLP; Gardner and Dorling, 1998) is a typical type of layer in feed-forward networks in which each node is connected to all the nodes in the next layer. Within each node in MLP, the input values are combined with weights and bias and then summed up before being passed to an activation function. The widely used activation functions include sigmoid, tangent hyperbolic (tanh; Schmidhuber, 2015), and rectified linear unit (ReLU; Nair and Hinton, 2021). The output *z* of a node in an MLP layer can be calculated as:


$$\begin{array}{l} z=\sigma \left(\overset{m}{\underset{i=1}{\sum }}w_{i}x_{i}+b\right) \end{array}$$

where *m* refers to the number of nodes in the current layer, *w* corresponds to the weights of all connections between the current node and nodes in the previous layer, *b* corresponds to bias, and σ corresponds to a non-linear activation function.

#### 2.1.2 Training an MLP

The weights of biases of the MLP are trainable parameters, which are optimized during the training process. Usually, those parameters are initialized with random variables close to zero. After the forward computation of the MLP, the loss function can be defined as the mean squared error (MSE) in single-class scenarios and cross-entropy in multi-class scenarios. Furthermore, the MLP weights can be learned in the training procedure by training with a basic error back-propagation technique for the loss function. Back-propagation is based upon an optimization algorithm using stochastic gradient descent (Bottou, 2012) with a pre-defined learning rate. During each round of computation, the values of the network parameters can be optimized by computing the gradient of the loss function with respect to each of them using the chain rule.

The input data of MLP is always separated into groups, and each group of samples is called a batch. The number of samples in the input group is referred to as a batch size. After all the data are trained, the procedure repeats a certain number of times, called an epoch number. Different from batch, an epoch indicates one iteration of the entire training dataset the ML model has completed. The number of entire iterations is named as epoch number. Except for the trainable parameters optimized during the training procedure, pre-defined parameters such as batch size, epoch number, or learning rate are fixed during training and are referred to as hyper-parameters.

#### 2.1.3 Overfitting and regularization

Overfitting occurs when a well-trained MLP fits accurately to the training data but performs poorly with the unseen test data. Especially in neuroimaging, the training sample size is limited (Mwangi et al., 2014), which is problematic for generalizing the findings to a clinical setting. There are two straightforward ways to address the overfitting problem: (i) simplifying the model, and (ii) increasing the training sample size. In addition, overfitting can also be addressed by adding regularization to the objective function. Those modifications, such as the well-known L1/L2 terms (Ridge and Lasso Regression), cause the model to be simpler during optimization but enhances the generalizability of unseen data (Eslami et al., 2021).

### 2.2 Baseline techniques for ASD classification

Machine learning techniques, such as SVM and MLP neural networks, performed well in the previous ASD classification studies (Bi et al., 2018; Chen et al., 2016; Chanel et al., 2016; Heinsfeld et al., 2017). To estimate the performance of the proposed domain adaptation approach, this study designated traditional SVM and MLP as baseline approaches. Specifically, a polynomial kernel was used in the SVM classifier, and the hyper-parameter *C* was set to 100. Likewise, the architecture of the baseline MLP method was the same as that of the VAE used in domain adaptation. The architecture has two layers, the first layer contains 200 nodes, and the second layer contains 500 nodes.

### 2.3 Participants and data

This study aimed to test the utility of the proposed domain adaptation model on the fMRI dataset. Particularly, this study used ABIDE resting-state fMRI data (Craddock et al., 2013). We used ABIDE I (Di Martino et al., 2014; released in August 2012) as the labeled dataset for supervised machine learning while ABIDE II (Di Martino et al., 2017; released in June 2016) as the unlabeled dataset for the semi-supervised machine learning algorithm. To investigate the domain adaptation effect of the proposed VAE-MMD model, we set ABIDE I as the source domain and ABIDE II as the target domain dataset. There were 998 subjects from 15 sites in the ABIDE I dataset and 623 subjects from 11 sites in the ABIDE II dataset.

ABIDE I fMRI data included 988 subjects from 15 different sites (and 18 cohorts since some sites had more than one cohort). The number distribution of subjects across multiple sites is shown in Table 1.

Our preprocessing followed widely adopted standard procedures in neuroimaging (Kalcher et al., 2012; Nyúl et al., 2000). The FMRI dataset was pre-processed using DPARSF (Chao-Gan and Yu-Feng, 2010). This involved the removal of the first five volumes: (i) slice timing correction, (ii) motion correction, (iii) co-registration to the standard MNI space, (iv) censoring of high motion volumes, and (v) regressing out nuisance variables (low-frequency drifts, mean global signal, motion parameters, and white matter and cerebrospinal fluid signals). Furthermore, voxel time series were temporally filtered with a 0.01–0.1 Hz bandpass filter. ABIDE II fMRI data included 623 subjects from 11 different sites. The pre-processing pipeline for this was identical to that used for ABIDE I and was performed in CONN software (Whitfield-Gabrieli and Nieto-Castanon, 2012).

**Figure 2:**
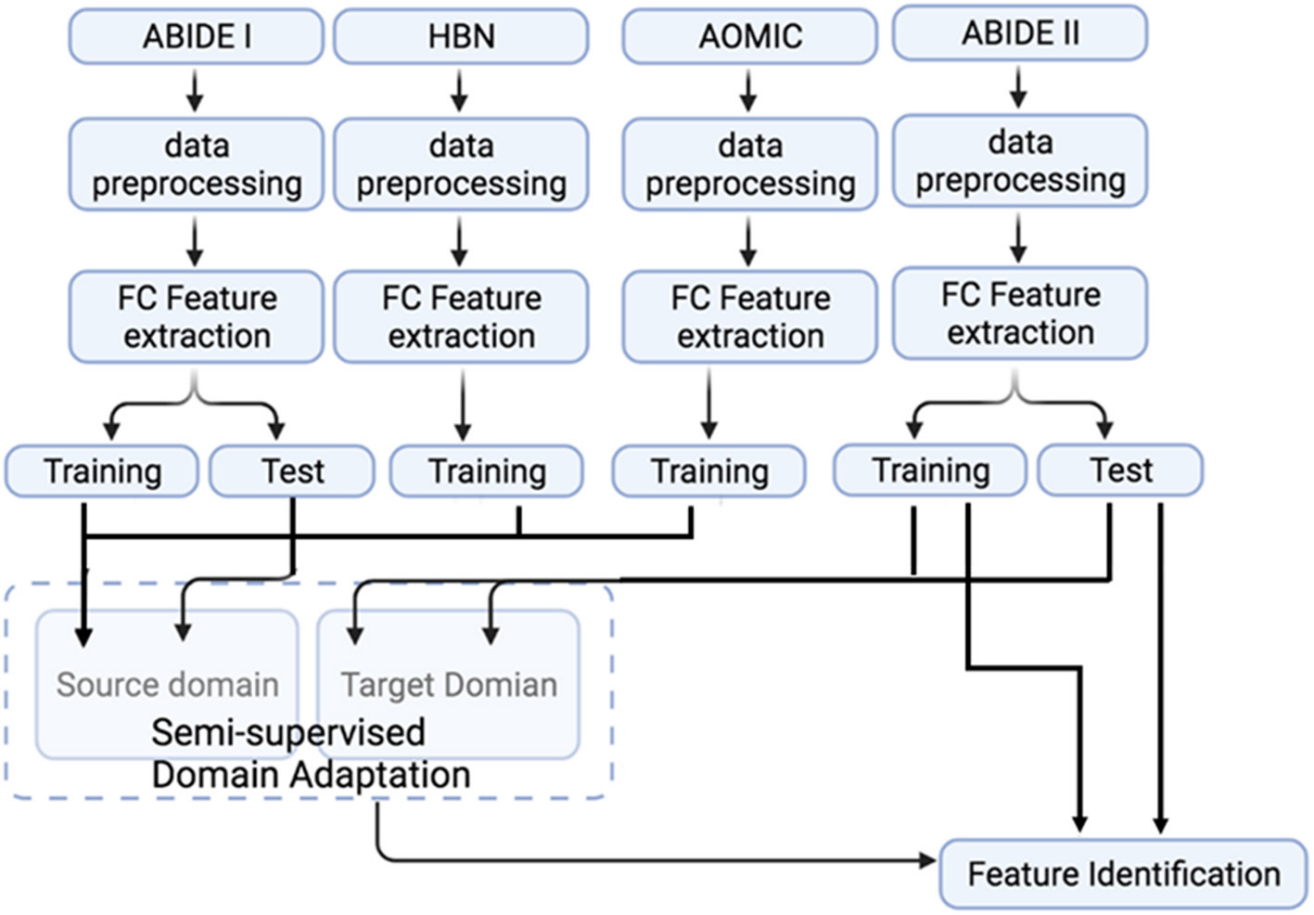
A flowchart representation of the complete processing and analysis of multiple datasets. The fMRI data from ABIDE I, ABIDE II, HBN, and AOMIC were subjected to identical data pre-processing and FC feature extraction. Source domain training and testing use ABIDE I data. In contrast, data from healthy subjects in AOMIC and HBN are used as additional training samples in the source domain to test the effect of source domain training sample size on domain adaptation performance. Target domain training and testing use ABIDE II data. Note that only the target domain ABIDE II data is used to identify features important for classification.

This study used two additional datasets: (i) the AOMIC (Snoek et al., 2021), and (ii) HBN datasets (Alexander et al., 2017). These datasets were used to test whether the model's generalizability can be further improved by augmenting the size of the source domain (adding more healthy control data). This study used AOMIC's raw forms, PIOP1 (*N* = 216) and PIOP2 (*N* = 226) datasets, instead of the pre-processed datasets. In addition, this study also used good quality MRI data from 1,385 subjects in HBN. The pre-processing pipeline for all the datasets was identical (see Figure 2). A schematic of the extended pipeline including ComBat harmonization is provided in the Supplementary Figure S1. The use of additional source domains such as AOMIC and HBN datasets were used to test whether increasing the size of the source domain or the number of source domain subjects improves domain adaptation. Specifically, the HBN dataset contains data from children. It may be relevant for domain adaptation when the target domain includes children's data (such as ABIDE or ADHD-200).

### 2.4. Feature extraction

We used the whole-brain cc200 atlas (Craddock et al., 2012) to reduce the dimensionality of the data. This atlas was generated using spectral clustering of resting state fMRI data of healthy subjects. Thus, the regions of interest (ROIs) in the atlas are said to be functionally homogeneous. Mean time series were extracted from 200 ROIs of the atlas. Subsequently, we estimated FC by computing the Pearson's correlation coefficient between each pair of time series. A vector of 19,900 individual features per subject was constructed by reshaping the upper triangle of the 200 × 200 connectivity matrix minus the diagonal. Only the upper triangle was considered since FC is a non-directional metric.

### 2.5 Domain adaptation VAE-MMD model with semi-supervised learning

**Figure 3:**
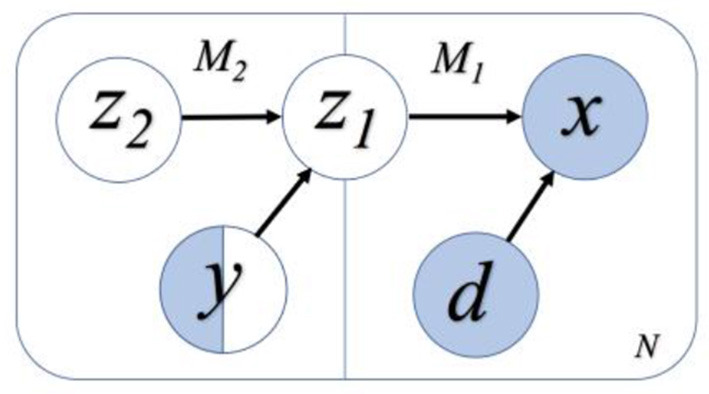
A flowchart representation of the semi-supervised learning model. White variables refer to the variables without input information, and blue variables refer to those with the input information. Only some of the labels of *y* are known, and hence *y* is half white and half blue. We assume the variables *z*_2_ and *y* are independent, while z_1_ and *d* are independent. Among them, *z*_2_, *y*, and *d* are independent variables, and *z*_1_ is dependent on *z*_2_ and *y*.

This study applies the semi-supervised VAE model that was initially proposed by the authors (Kingma et al., 2014) with unsupervised learning. The proposed model consists of a generative model *p*_θ_(*x*|*z, d*) and an inference model *q*_ϕ_(*z*|*x, d*), where *z* is the latent variable representation, *x* is the input data, and *d* is the domain variation that is desired to remove. Moreover, θ and ϕ are the trainable parameters of the generative model and inference model, respectively. For semi-supervised classification, this study aims to construct latent variable *z*, which has maximum information about the observed label *y*, while excluding the information about the nuisance domain variable *d*. It is achieved by adding an additional model in the generative model to correlate latent features to the classification task (Louizos et al., 2016). The schematic of this model is shown in Figure 3, where the invariant feature in the first model, M1, is referred to as *z*_1_. M1 generates *x* as *x* ~ *p*_θ_(*x*|*z*_1_, *d*), and M2 generates domain invariant variable *z*_1_ as *z*_1_~ *p*_θ_(*z*_1_|*z*_2_, *y*). *y* is a categorical variable that denotes the label of the data point *x* and *z*_2_ encodes the variation on *z*_1_ that is independent to *y*. Thus, for the N labeled data points and M data points without labels (i.e., unlabeled data), the objective function of VAE becomes:


$$\begin{array}{l} {ℱ}_{VAE}\left(\phi ,\theta ;x_{n},x_{m},d_{n},d_{m},y_{n}\right)=\sum_{n=1}^{N}{ℒ}_{s}\left(\phi ,\theta ;x_{n},d_{n},y_{n}\right)\\ \;+\;\sum_{m=1}^{M}{ℒ}_{T}\left(\phi ,\theta ;x_{m},d_{m}\right)\\ \;+\;\alpha \sum_{n=1}^{N}E_{q\left(z_{1n}|x_{n},\;d_{n}\right)}[-logq_{\phi }(y_{n}|z_{1n})] \end{array}$$

where the first and second terms denote the lost functions from the labeled and unlabeled data. In addition, the label predictive distribution *q*_ϕ_(*y*|*z*_1*n*_) only contributes to the unlabeled data in the second term. Therefore, we compensate for this by adding a regularization term with a weight coefficient α to ensure that *q*_ϕ_(*y*|*z*_1*n*_) is learned from both labeled and unlabeled data. Finally, increasing α results in more purely discriminative learning in the generative model.

In the VAE inference model, we assume that variables *z*_1_ and *d* are statistically independent of each other so that the marginal posterior distribution *q*(*z*_1_|*d*) is equal to zero. However, the independence relationship may fail because of the correlation between *y* and *d*. We apply an additional MMD regularization term to penalize this situation.

In the MMD definition, the divergence between two distributions is calculated as the distances between mean embeddings of features (Tzeng et al., 2014). Let *k* be a continuous, bounded, positive semi-definite kernel and *H* be the corresponding reproducing kernel Hilbert space (Gretton et al., 2012), which are reduced by the feature mapping from *X* to *H*. The MMD of distributions *p*_*x*_(*x*) and *p*_*y*_(*y*) is defined as follows:


$$\begin{array}{l} MMD\left(p_{x},p_{y}\right)=\;||E_{x\sim p_{x}}\left[\varphi \left(x\right)\right]-\;E_{y\sim p_{y}}\left[\varphi \left(y\right)\right]|{|}_{H}^{2} \end{array}$$

In the VAE model, an additional MMD regularization term was applied to enforce the model to match the source and target domain marginal posterior distributions of latent variables *q*(*z*_1_|*d* = 0) and (*z*_1_|*d* = 1). So, the MMD term is determined as:


$$\begin{array}{l} \ell_{MMD}\left(Z_{1,d=0},Z_{1,d=1}\right)=\;\|E_{\tilde{p}\left(x|d=0\right)}[E_{q\left(z_{1}|x,d=0\right)}\left[\varphi \left(z_{1}\right)\right]]\\ \;-\;{E_{\tilde{p}\left(x|d=1\right)}[E_{q\left(z_{1}|x,d=1\right)}\left[\varphi \left(z_{1}\right)\right]]\;\|}_{H}^{2} \end{array}$$

Where *d* is the domain nuisance variable. Finally, adding the MMD penalty term into the lower bound of the aforementioned VAE, the proposed model becomes:


$$\begin{array}{l} \;{ℱ}_{MMD-VAE}\left(\phi ,\theta ;x_{n},x_{m},s_{n},s_{m},y_{n}\right)=\\ {ℱ}_{VAE}\left(\phi ,\theta ;x_{n},x_{m},s_{n},s_{m},y_{n}\right)-\beta \;\ell_{MMD}\left(Z_{1,s=0},Z_{1,s=1}\right) \end{array}$$

where β denotes the regularization coefficient in domain adaptation, increasing β results in more domain confusion regularization compared to the classification loss. Both α and β are hyper-parameters that control the trade-off between classification loss and domain confusion loss, which are optimized through training and validation.

**Figure 4:**
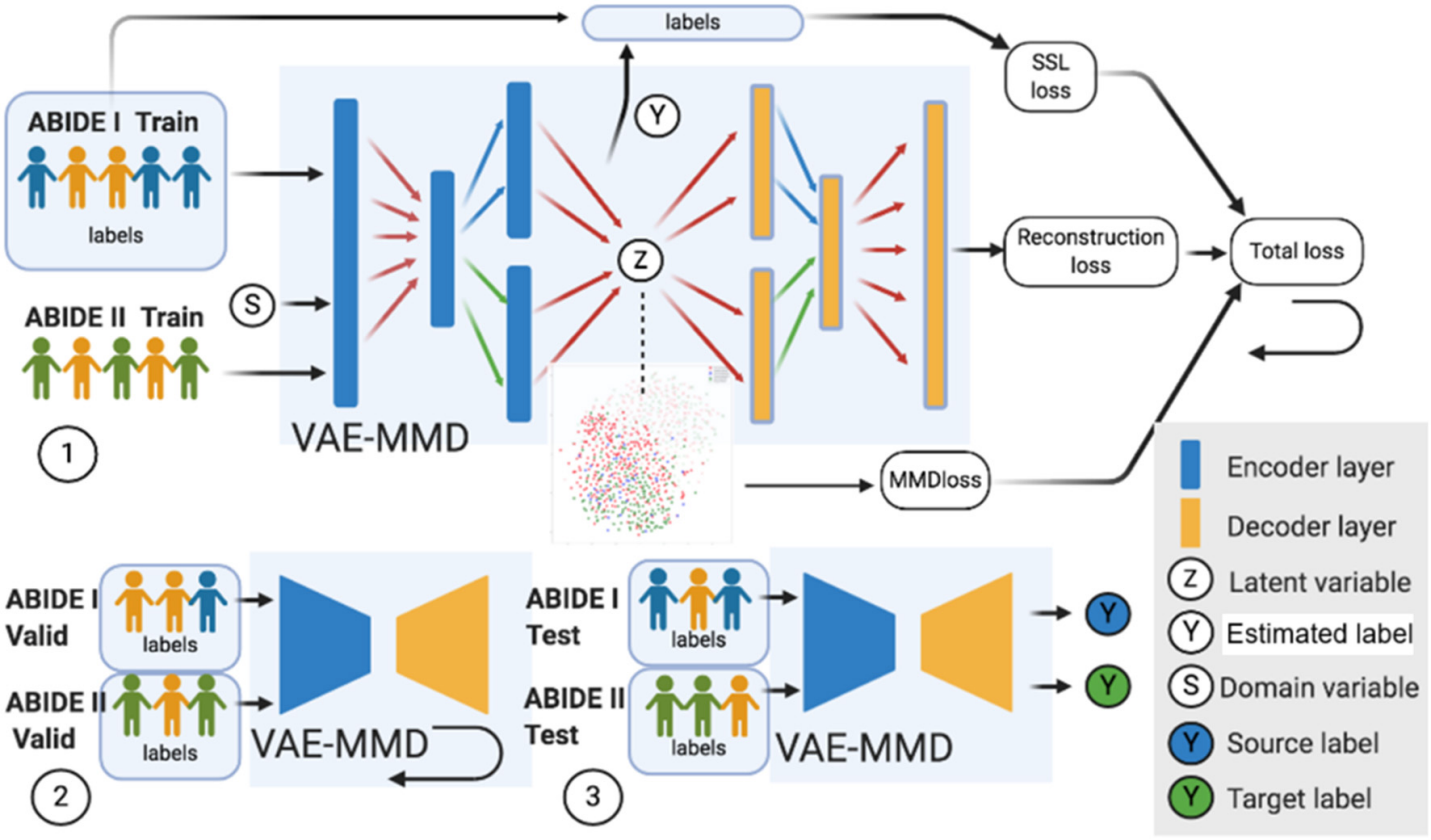
Three major steps in the VAE-MMD model. (1) For training, we input both ABIDE I with labels and ABIDE II (without labels) training datasets into the VAE-MMD model. The original t-SNE figure and domain adapted t-SNE figure were generated at the beginning and last iteration of this process. The total loss was constructed by semi-supervised learning loss, reconstruction loss, and MMD loss. (2) For validation, ABIDE I and ABIDE II validation datasets were used for fine-tuning the hyperparameters α and β. (3) For testing, ABIDE I and ABIDE II test datasets were used in testing the model to evaluate the model's performance. Subjects in orange represent healthy controls in ABIDE I and ABIDE II data, subjects in blue represent ASD subjects in ABIDE I, and subjects in green represent ASD subjects in ABIDE II.

**Figure 5:**
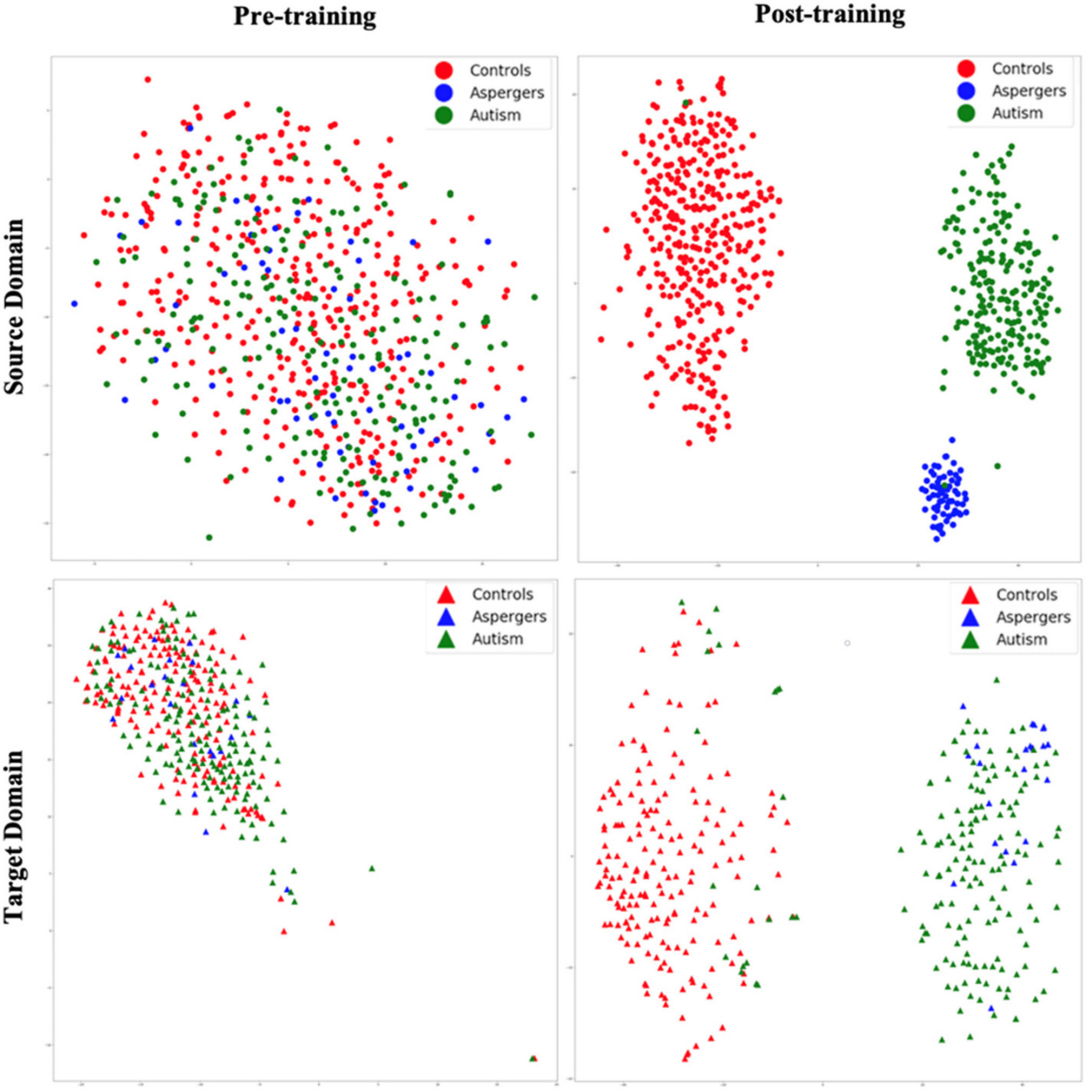
t-SNE visualization of latent feature spaces for VAE-MMD domain adaptation model. High-dimensional data is reduced to two dimensions for visualization through t-SNE. **Left panel:** clustering before training; **right panel:** clustering after training; **top panel:** source domain; **bottom panel:** target domain. Red color corresponds to controls, whereas blue and green colors correspond to Asperger's and autism patients, respectively. Circle marks correspond to the source domain, and triangle marks correspond to the target domain.

The datasets input to the semi-supervised learning model are illustrated in a flowchart, described in Figure 2, and the entire framework is shown in Figure 4. In the training model (#1 in Figure 4), we input both ABIDE I with labels and ABIDE II without labels as training datasets into the VAE-MMD model. After domain adaptation, the original t-distributed Stochastic Neighbor Embedding (t-SNE) figure and the corresponding t-SNE figures (Figure 5) were generated at the beginning and last iteration of this process. Moreover, t-SNE (van der Maaten and Hinton, 2008) is a dimension reduction technique to visualize the group-wise separation of features in latent space and visually assess domain adaptation's efficacy.

In the validation model (#2 in Figure 4), ABIDE I and ABIDE II validation datasets were used to fine-tune the hyperparameters α and β, and ABIDE I and ABIDE II test datasets were used to measure the model's performance (#3 in Figure 4). We used accuracy measure to evaluate the training, validation, and testing models to better understand the model performance.

### 2.6 Model setup

MLPs (Nozais et al., 2021) work well on vector inputs, while CNN's perform better on natural images. Since functional connectivity inputs can be vectors and are not natural images, this is why this study used MLPs. The first layer is constructed as the latent-feature discriminative model (M1) in the encoder, and the second layer is built as a generative semi-supervised model (M2) in a stacked architecture. M1 refers to the first layer and M2 to the second layer in the encoder of #1 in Figure 4. The dimension of latent features in the first and the second encoding layers was equal to 2,000 and 1,000, respectively. The learning rate was set to 0.0001, and each neural network layer used ReLU as an activation function (Pedamonti, 2018). The epoch number was equal to 50, and the number of batches was 20. The code was constructed in Python programming languages and the Theano library.

We set ABIDE I as the source domain dataset and ABDIE II as the target domain dataset. We aimed to reduce the non-neural differences in data characteristics between the two domains. The ABIDE I dataset was split into 673/157/158 subjects as training, validation, and test datasets, respectively. The labeled data was used in the training and validation datasets, and these datasets were used in a cross-validation framework for fine-tuning hyperparameters. The ABIDE II dataset was split into 371/126/126 subjects as training, validation, and test datasets, respectively.

To avoid data leakage, we strictly separated the datasets for training, validation, and testing. Specifically, the VAE and VAE-MMD models were pretrained using ABIDE-I (source domain) and the training set of ABIDE-II (target domain), and additional healthy control data from the HBN and AOMIC datasets as part of transfer learning. The ABIDE-II test set (126 subjects) was completely held out during model development and hyperparameter tuning. The validation set (126 subjects from ABIDE-II) was used solely for tuning model parameters, and no information from the test set was used at any point during training or model selection. To further prevent overfitting, model training was conducted using five-fold cross-validation on the combined training data.

### 2.7 Transfer learning

Transfer learning (TL; Ghafoorian et al., 2017) is a technique that applies knowledge learned from one domain and one task to another related domain and/or another task (Heinsfeld et al., 2017). HBN and AOMIC data were included as additional source domain data to improve generalizability, address overfitting, and increase sample size in the source domain. The labels of these two datasets are all healthy controls that were used during training. The number of batches of HBN and AOMIC was equal to that of the ABIDE dataset to be trained simultaneously. The divergences of these two datasets to the target domain data were also optimized during training, the same as ABIDE I data in the source domain.

### 2.8 ComBat harmonization

We used the publicly available MATLAB toolbox (Fortin and Foran, 2021) to achieve ComBat harmonization and used default options. Finally, we separated harmonized data into training and testing datasets and input it into the deep learning model to evaluate classification performance. This metric was compared with that obtained from the VAE-MMD model.

### 2.9 Model estimation

The performance of the models was estimated at three levels. First, visualization of the separation of features in latent space of the VAE-MMD model was realized using t-SNE plots (van der Maaten and Hinton, 2008). Second. Kullback–Leibler (KL) divergence was used to characterize the separation of features analytically. In other words, KL divergence was used to quantify the difference between the target and source domains analytically. Third, the models' performance was characterized by accuracy and F1 score. We compared the classification accuracy among multiple machine learning models with the same datasets. The models included SVM, MLP, VAE, VAE, and MMD combination (VAE+MMD), VAE and ComBat harmonization combination (VAE+ComBat) and domain adaptation combined with transfer learning (VAE+MMD+TL). The benchmark for harmonization is VAE+ComBat, while VAE+MMD and VAE+MMD+TL show the improvements obtained by MMD and TL over ComBat. Additional combinations involving both ComBat and domain adaptation (VAE+MMD+ComBat and VAE+MMD+ComBat+TL) are presented in the Supplementary material for the interested reader.

Accuracy represents how close the prediction comes to the true values. It is determined by the number of correct predictions divided by the total number of predictions. Due to the class imbalance in the test dataset, we used F1-score to combine both precision and recall of each class, and the F1-score (Pedregosa et al., 2011) can be calculated as follows:


$$\begin{array}{l} F1\;=\;2\times \frac{Precision\times Recall}{Precision+Recall} \end{array}$$

### 2.10 Feature identification

The first encoding layer is the most interpretable because the weights between each node of the encoding layer to the next hidden layer are considered as learned features (Montavon et al., 2018; Kim et al., 2016). This study also analyzed the weights from the encoding layer to the next hidden layer to explain the importance of features in the classification as reported in the previous studies (Guo et al., 2017; Vakli et al., 2018). Furthermore, we applied permutation testing to identify the statistically significant features. Once the model was trained, the weights assumed values accordingly during the training process. At the end of the training process, each weight had a mean value calculated over all iterations of training. This mean weight represented the “importance” of the corresponding feature in the input weight vector of size 1 × 19,900. During permutation, the order of the input vector was randomly shuffled, and the training process was repeated after each shuffle. The mean weight obtained during each permutation corresponded to the importance of different features in different permutations. The distribution of mean weights obtained across permutations (1,000 of them) represented a null distribution of the hypothesis that all features were significantly important. The *p*-value of node A was calculated by the number of mean weight values greater than the true value, divided by the total number of mean weight values. Moreover, the *p*-value was corrected for multiple comparisons using the false discovery rate (FDR) method at 5%. The *p*-value can be calculated as follows:


$$\begin{array}{l} P_{A}\;=\;\frac{Num_{greatTrue}}{{Num}_{permutation}}\;\times \;100\% \end{array}$$

the *Num*_*greatTrue*_ refers to the number of permutations where the mean value of weights was greater than the true value, and *Num*_*permutation*_ refers to the total number of permutation tests (=1,000). The permutation testing procedure was identical for all of the proposed models.

## 3 Results

### 3.1 Domain adaptation

Figure 5 shows t-SNE visualizations of the latent feature space in both the source (top panel) and target domains (bottom panel), prior to (left panel) and after (right panel) training. Before training, there was little separation between the diagnostic groups in both the source and target domains. However, after the training process, a clear separation of the diagnostic groups in latent space emerged in the source domain. This is transferred to the target domain as a visible separation between diagnostic groups [with some exceptions, especially between autism and Asperger's (Note: Although the term “Asperger's syndrome” is no longer used in the DSM-5 (2013) and is now categorized under Autism Spectrum Disorder (Level 1), we retain the original label in this manuscript to reflect the diagnostic terminology used in the ABIDE dataset at the time of data collection.)] can be observed. Thus, the results revealed that even with high dimensional input data, the VAE-MMD model reduced the distance between the data points from the same class but different domains in latent space.

**Figure 6:**
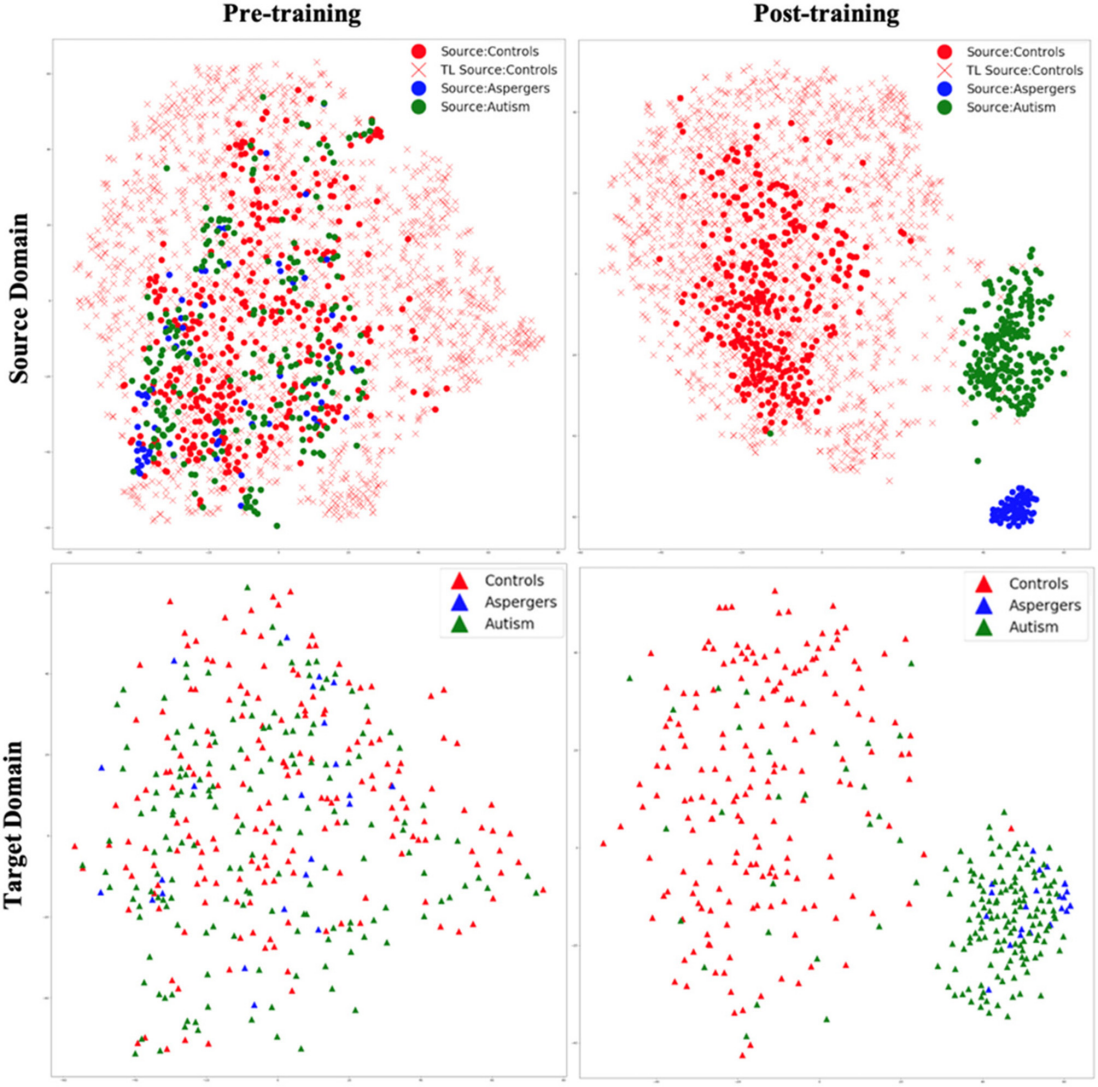
t-SNE visualization of latent feature spaces for VAE-MMD domain adaptation model, with transfer learning from additional healthy control data in the source domain drawn from HBN and AOMIC datasets. **Left panel:** clustering before training; **right panel:** clustering after training; **top panel:** source domain; **bottom panel:** target domain. Red color corresponds to controls, whereas blue and green colors correspond to Asperger's and autism patients, respectively. Circle marks correspond to ABIDE-I subjects in the original source domain; cross marks correspond to additional HBN and AOMIC healthy control subjects in the source domain, and triangle marks correspond to the target domain.

Healthy control subjects from HBN and AOMIC datasets were given additional source domain data as input. Since learning about healthy control subjects in one domain (HBN and AOMIC) is “transferred” to another domain (ABIDE), this specific case of domain adaptation is referred to as “transfer learning.” The t-SNE embedding (Figure 6) shows the latent feature distributions for the VAE-MMD domain adaptation model, with transfer learning from additional healthy control data in the source domain drawn from HBN and AOMIC datasets. As with the earlier case, there was a little separation between groups before training, partly because the non-neural inter-site differences drown out the inter-group neural differences. After training, it can be observed that separation between groups is near perfect in the source domain and visible in the target domain (with some missed assignments to the wrong cluster).

Comparing Figures 5, 6, it is noteworthy that including additional healthy control data in the source domain from HBN and AOMIC datasets expanded the reach of the healthy control cluster in both the source and target domains. This implies that the model captured a larger variance in the healthy population and became more generalizable in the target domain, as evidenced by improved target domain accuracies presented in the next section. As elaborated in the discussion, we hope that with more publicly available healthy control data input into the proposed model in the future, the model's generalizability can be further improved, leading to more realistic separation boundaries between groups. Finally, this can improve performance on unseen test data in the target domain.

### 3.2 Classification accuracy

**Figure 7:**
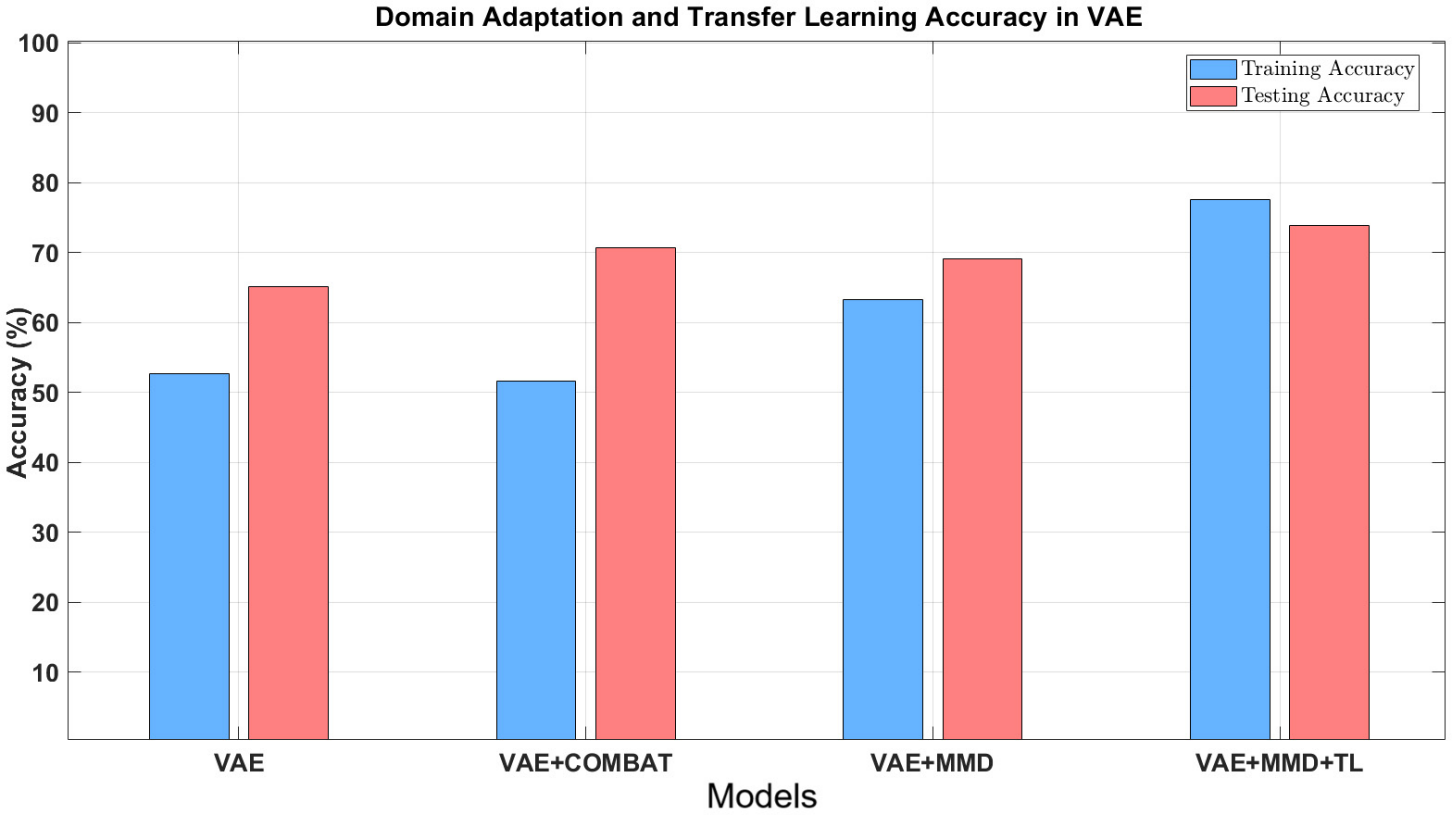
The classification accuracy using different approaches combined with domain adaptation. Blue bars refer to the training accuracy in the target domain, and red bars refer to the testing accuracy in the target domain.

Table 2 shows the accuracy and F1-score from the VAE-MMD model (i.e., domain adaptation) when combined with other strategies, such as transfer learning (TL) from HBN and AOMIC datasets, as well as statistical harmonization (ComBat). Moreover, the results from the baseline methods SVM and MLP are included. It is worth mentioning that the baseline methods did not perform well because of the domain shift between source domain data and target domain data. Compared to the baseline methods, all other techniques containing VAE obtained better results. All models have almost 100% training accuracy in the source domain, which is not surprising since training accuracy tends to be saturated. However, the testing accuracy of the source domain is poor, given the inability to generalize based just on the source data. This agrees with prior results of standard machine learning methods for neuroimaging data (Lanka et al., 2020). In addition, MMD-based domain adaptation enhances the accuracy during target domain training, but the final F1-score on the target test dataset remains comparable to that achieved by the ComBat harmonization technique (69.05% vs. 70.63%). For the three classes in the target domain dataset (controls, autism, and Asperger's), MMD domain adaptation can increase accuracy by 4%−10% without using target domain labels. Incorporating transfer learning using additional healthy control datasets further boosts accuracy (73.81%), outperforming ComBat and demonstrating the benefits of combining domain adaptation with transfer learning (see Table 2 and Figure 7). Combining transfer learning with ComBat harmonization further improves performance, increasing accuracy from 73.81% to 75.4% (see Supplementary Table S1 and Supplementary Figure S2).

**Table 2:** Classification results were obtained by combining domain adaptation (VAE-MMD) with other strategies such as transfer learning (TL) and statistical harmonization (ComBat).

**Classification accuracy**	**Source training (%)**	**Source test (%)**	**Target training (%)**	**Target test (F1-score)**
SVM	–	–	–	49.32% (0.32)
MLP	–	–	–	62.66% (0.30)
VAE	99.97	64.56	52.67	65.08% (0.27)
VAE+COMBAT	100	60.76	51.56	70.63% (0.29)
VAE+MMD	99.67	50.94	63.29	69.05% (0.35)
VAE+MMD+TL	100	60.76	77.61	73.81% (0.38)

For each of the training and testing datasets (test sample size equal to 126), we compared the classification accuracies from source and target domains. In addition, we used SVM, MLP, and VAE trained on source domain data and tested on target domain data. The last column shows the F1 score of each approach. Domain adaptation was not applied with SVM and MLP, so there was no source training, source test, and target training classification accuracies.

However, given the smaller number of samples from Asperger's and its similarities with autism, three-way classification in ABIDE is challenging. Although cross-validation accuracies above chance (which is 33%) have been reported before, accuracy in independent test datasets rarely exceeded 70% (Abraham et al., 2017; Li et al., 2018; Lanka et al., 2020). If we included AOMIC and HBN datasets into the source domain, accuracy further increased to 73.81% due to transfer learning, demonstrating that there is scope within the domain adaptation framework to improve the accuracy by adding more data. Considering differences in data distribution between AOMIC and HBN datasets (Table 1), this study also investigated how much performance improvement was caused by HBN and AOMIC separately. Supplementary Table S2 shows the accuracy results separately from HBN and AOMIC datasets in the VAE+ComBat+MMD+TL framework.

### 3.3 Feature identification

**Figure 8:**
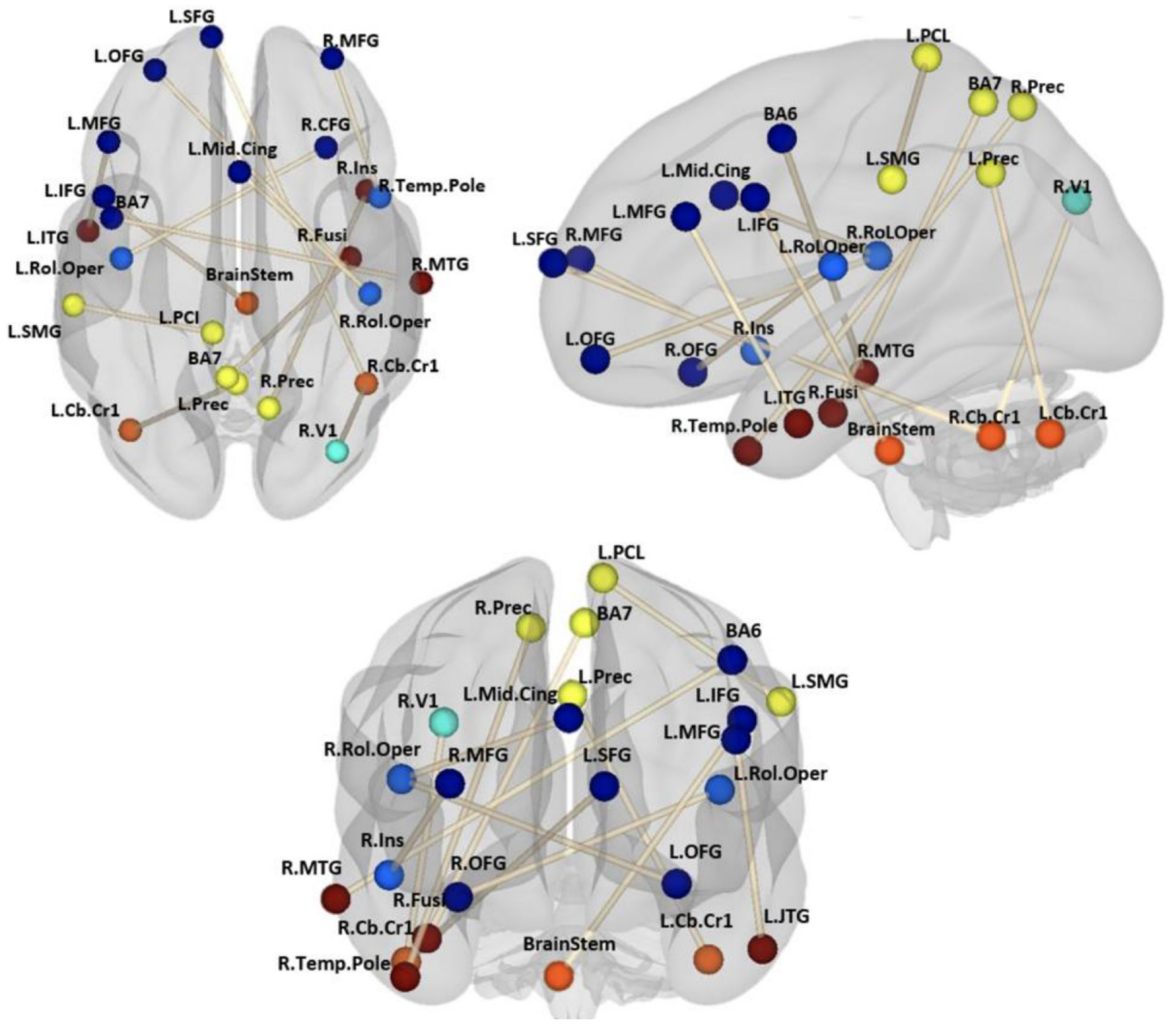
FC features found to be important for classification using our VAE+MMD+TL model with the highest target testing accuracy. The figure shows coronal, sagittal, and axial views of connections with colormap. The colors represent different lobes: dark blue: frontal lobe; light blue: insular lobe; cyan: occipital lobe; yellow: parietal lobe; orange: subcortical; red: temporal lobe.

Figure 8 shows the importance of features for the classification using the VAE+MMD+TL model. These paths also happen to be significantly weaker (p < 0.05, FDR corrected; Benjamini and Hochberg, 1995) in ASD and Asperger's than in healthy controls. Except for the local connection of supramarginal gyrus to postcentral gyrus in the parietal lobe, most of the paths were cross-network and cross-lobe connections, including middle frontal gyrus to inferior temporal gyrus, and BA6 to middle temporal gyrus in the fronto-temporal network, orbito-frontal gyrus to rolandic operculum in the fronto-insular network, and right precentral to right temporal pole in the temporo-parietal network. Most of the affected regions in the frontal lobe were left-lateralized.

The significant connectivity patterns we identified have been previously reported in several studies related to deficits in social, behavioral, and communicative functioning in individuals with ASD. For example, Noonan et al. (2009) reported hyperconnectivity between the fusiform gyrus, an area associated with decoding social cues such as facial expressions (Delbruck et al., 2019), and the superior parietal lobule (BA7) in individuals with ASD. This altered connectivity may reflect difficulties in integrating visual information with attentional processes, which could contribute to impairments in social perception and interaction observed in ASD. We identified this connection as well (shown in Figure 8). Yoon et al. (2022) reported decreased functional connectivity between the middle frontal gyrus (MFG) and the inferior temporal lobule (ITG) in children with ASD. We also report this as an important connection in Figure 8. Connectivity disruption between MFG and ITG was significantly correlated with clinical measures such as social communication and awareness scores from the Social Responsiveness Scale (SRS). Additionally, Yoon et al. found reduced local efficiency in these regions, suggesting impaired information integration within the social brain network in ASD. Connections between the frontal cortex and insular networks were also observed (e.g., R OFC and RolOper; R MFG and R Ins, as shown in Figure 8). Altered connectivity among insular subregions has been implicated in the degradation of emotion regulation abilities in individuals with ASD (Ong and Fan, 2023; Taylor et al., 2009; Zhao et al., 2022), while frontal regions such as the OFC and MFG have been associated with social impairments in ASD (Ong and Fan, 2023). Disruptions in these fronto-insular pathways may therefore contribute to the emotional and behavioral deficits characteristic of the disorder (Ong and Fan, 2023). Altered connectivity between BA6 and the middle temporal gyrus (MTG) within the fronto-temporal network has been implicated in ASD. Xu et al. (2020) identified distinct subregions within the MTG and found that individuals with ASD exhibited hypoconnectivity between these MTG subregions and various frontal areas associated with motor planning and social cognition. Similarly, Cheng et al. (2015) reported reduced connectivity between the MTG and regions involved in emotion processing and theory of mind, such as the ventromedial prefrontal cortex, further supporting the role of disrupted fronto-temporal interactions in the social and cognitive impairments observed in ASD.

## 4 Discussion

Large public databases, such as ABIDE (Autism Brain Imaging Data Exchange), have aided deep learning models for diagnostic classification with potential applications in AI-assisted clinical decision systems. However, such large public databases have been assembled *post-hoc* and contain different non-neural variabilities sources, such as different sites using different scanners and protocols, which degrade the performance of deep learning as well as traditional machine learning models (Li et al., 2018; Lanka et al., 2020). To address this, this study proposed and implemented a domain adaptation framework by employing a VAE-MMD deep learning model using ABIDE I as the source domain and ABIDE II as the target domain. We demonstrated improved classification performance in the target domain by utilizing the source domain's knowledge and making data distributions in the source and target domains as similar as possible (Li et al., 2020; Zhou et al., 2018). When used in combination with domain adaptation and transfer learning, the ComBat statistical harmonization (Fortin et al., 2018, 2017, 2016) further improved the classifier's performance (see Supplementary material). Finally, we also showed that additional transfer learning from HBN and AOMIC datasets improved the classification accuracy.

To analyze the effect of domain shift and adaptation, we compared the performance of several models. The baseline VAE model, trained and evaluated solely on ABIDE-II, achieved an F1-score of ~68.06%. In contrast, training the same VAE model on ABIDE-I and testing it directly on ABIDE-II resulted in a lower F1-score of 65.08%, likely due to domain shift caused by differing data distributions across sites. This highlights the challenges of generalizing across datasets without adaptation. Incorporating a Maximum Mean Discrepancy (MMD) loss into the VAE framework led to improved performance (F1-score: 69.05%) by explicitly aligning latent feature distributions between the source and target domains. This result demonstrates that domain-invariant representations learned via MMD can enhance generalization to unseen data.

Even with high dimensional input features, the VAE-MMD model was able to project data points from different domains from the same class into a closed latent space. This study demonstrates that the proposed approaches can improve target domain classification when used independently. When these models were combined, the accuracy was better than the models' individual performance. Specifically, Figure 7 and Table 2 showed that learning from labeled training data in the source domain improved dramatically with domain adaptation and ComBat-harmonization, with the same trends seen in the target domain with unlabeled data, but to a lesser extent (Kouw and Loog, 2021). Compared to these two methods (ComBat harmonization and domain adaptation approach), ComBat required minimal hardware and time to complete the harmonization. In contrast, the deep learning model required more time due to the fine-tuning of hyper-parameters. It remains to be seen whether the improvement in performance expected by including larger datasets in the deep learning framework will justify the additional computational complexity compared to statistical ComBat harmonization.

This study used a three-class classification approach (Autism, Asperger's syndrome, and Controls). The Asperger's population is more similar to autism than healthy controls (Duffy et al., 2013). However, it is still distinctly separate from typical autism across behavioral, cognitive, and neural domains (Faridi and Khosrowabadi, 2017). However, several studies prefer to perform two-way classification between controls and ASD; some studies perform three-way classification (Wang J. et al., 2020). This study reported the performance of three-way classification in the relatively larger ABIDE dataset and obtained a modest performance. However, the relatively good three-way classification accuracy of deep learning vs. traditional machine learning models in the case of ASD (over 70% accuracy) has been reported in smaller datasets (*N* = 114; Isam et al., 2021). This study found that the VAE+MMD+TL approach outperformed SVM and MLP methods by enhancing the classification of the Asperger's class from < 10% to about 60%. One of the three-way ASD classification studies (Wang J. et al., 2020) also applied a domain adaptation approach and used functional connectivity as an input feature. Still, the authors reported < 60% accuracy in ASD classification. Thus, compared to other three-way ASD classification studies, the proposed approach obtained a high accuracy of over 75% on the test dataset (see Table 2). For comparison, the two-way classification results for our model are included in the Supplementary material.

This study also utilized a semi-supervised domain adaptation approach that combined the advantages of UDA and SDA. Moreover, the proposed approach is the first method to utilize such a UDA framework in a classification task of a neurodevelopmental condition without the annotated labels in the target domain (Choudhary et al., 2020). According to the best of our knowledge, this research is also the first study that used t-SNE as a visualization method in the prediction of neurodevelopmental conditions. The proposed approach provided higher accuracy in ASD classification than other SDA studies, such as the research by Shi et al. (2021). The authors (Shi et al., 2021) trained the three-way decision domain adaptation classifier with the MMD mod, then applied it to FC from the ABIDE dataset and obtained around 71% accuracy. The researchers used propagated pseudo labels to target domain data trained by an SVM classifier, which did not benefit from a deep learning classifier to handle high-dimensional data as in our model. Another study (Wang M. et al., 2020) treated one individual site as a target domain and all other sites as source domains. Then, a common low-rank latent representation was constructed across the source and target domains, obtaining 60% to 70% accuracy. Thus, the proposed approach (at 75.4% accuracy) yields superior performance over these state-of-the-art domain adaptation methods applied to ASD prediction.

Since domain adaptation improved target domain test accuracy, it raises the possibility that additional data in the source domain may further improve classification performance. Therefore, we augmented the source domain with additional healthy control data from HBN and AOMIC datasets. From Table 1, it is clear that AOMIC has a higher proportion of females and is older in mean age than the other three datasets. Despite this, we chose AOMIC, intending to improve the generalizability of the classifier (by exposing the classifier to different age/gender mixes). The results from separate datasets are shown in Supplementary Table S2; HBN provided slightly better performance than AOMIC because it has similar age and gender to ABIDE. Combining both AOMIC and HBN datasets, transfer learning from these datasets to discrimination in the target domain showed improved performance. Furthermore, If additional data can improve performance further, it opens up the possibility of building truly generalizable classifiers at scale. This is an essential step in making machine learning models based on neuroimaging data relevant for AI-based diagnostic support in the clinic, rather than being a purely academic tool (which it is currently) for understanding discriminative features of brain function in mental disorders.

To further evaluate the contribution of each component, we conducted a series of ablation comparisons. The baseline VAE model trained on ABIDE-I and tested on ABIDE-II achieved a test accuracy of 65.08%, indicating a strong domain shift. Incorporating MMD (VAE+MMD) improved accuracy to 69.05%, while adding ComBat alone (VAE+ComBat) raised performance to 70.63%, suggesting its effectiveness in reducing site-related variance. When both MMD and ComBat were combined (VAE+MMD+ComBat), accuracy reached 74.6%. Adding transfer learning from HBN and AOMIC (VAE+MMD+TL) led to 73.81%, highlighting the benefit of additional healthy control data. The full model combining all three elements—MMD, ComBat, and transfer learning—achieved the highest accuracy of 75.4%. These results support the additive benefit of each module. We did not include a VAE+TL condition in this study, as it addresses a distinct research question beyond the scope of this manuscript.

## 5 Limitations and future work

This study contains important limitations. First, this study analyzed the weights from the encoding layer to the next hidden layer to explain the importance of features in the classification. While this is based on prior research (Guo et al., 2017; Vakli et al., 2018), one could optimize this choice further by exploring best interpretability algorithms for the proposed machine learning model and representations of domain invariant features. Second, we acknowledge that our study does not explore the limits of performance improvements from domain adaptation and transfer learning. Determining the point at which additional data ceases to yield further benefits would likely require access to significantly larger datasets comprising thousands of subjects. Currently, among publicly available datasets, only large-scale initiatives such as the UK Biobank would be suitable for such an analysis. Other neurodevelopmental cohorts, such as the NKI-Rockland Sample (Nooner et al., 2012) and the Philadelphia Neurodevelopmental Cohort (Satterthwaite et al., 2016), may also be considered for extending training and improving generalizability. We plan to incorporate data from the UK Biobank (Miller et al., 2016) in future research to address this limitation. Third, how dependent is the performance of the framework on the inherent heterogeneity of the (i) sample, (ii) disorder, and (iii) data acquisition and pre-processing strategies need to be investigated further. Fourth, the class imbalance issue was not examined to enhance the performance of the multi-class approach. For example, we observed that classification performance for the Asperger's class in the target domain was lower compared to the control and autism classes, consistent with the limited separation observed in the t-SNE visualizations (Figures 5, 6). This suggests that distinguishing Asperger's syndrome from autism remains challenging and represents a limitation of the current model, which may be driven by the lower sample size for this group. Techniques such as synthetic oversampling (Chawla et al., 2002) have been proposed to mitigate class imbalance and could be explored in future work. Fifth, our paper does not report statistical comparisons between model accuracies, such as *p*-values or confidence intervals. Since the model was trained with five-fold cross-validation, the effective sample size for statistical testing is very small (*N* = 5). In such settings, *p*-values can be unstable or misleading, and confidence intervals may appear artificially narrow or overconfident due to the limited number of folds. We also note that many prior works in neuroimaging and deep learning (Alzakari et al., 2025; Farooq et al., 2023; Parisot et al., 2018; Shao et al., 2021; Yang et al., 2021) evaluate model performance based on the mean and standard deviation across folds or repetitions without reporting *p*-values. We believe this approach offers a fair assessment of the model's effectiveness given the statistical limitations of five-fold cross-validation. Finally, this study presents t-SNE plots as a qualitative visualization of how the latent features separate diagnostic groups and align domains before and after training. We did not include quantitative clustering metrics such as the Silhouette Score or Davies–Bouldin Index because our primary objective was classification rather than unsupervised clustering, and these metrics do not directly measure class label separability or domain alignment in supervised settings. In addition, clustering metrics may be unreliable when applied to t-SNE-reduced spaces, which are optimized for visualization rather than preserving global data structure. Instead, we relied on downstream classification performance (accuracy and F1-score) on a held-out test set as a more direct evaluation of the model's ability to learn discriminative and domain-invariant representations.

## 6 Conclusion

In conclusion, the results of this study demonstrate that domain adaptation and transfer learning, when used in combination, outperforms ASD classification in test data as compared to baseline statistical harmonization methods of multi-site data such as ComBat. The domain adaptation VAE-MMD model is robust against sources of data distribution divergence, such as inter-site differences in data acquisition parameters and scanner models. By demonstrating that augmenting the source domain with additional data leads to improved target domain accuracy due to transfer learning, this work opens the possibility of further improving the proposed model by utilizing the ever-increasing amount of healthy control neuroimaging data in the public domain.

## Data Availability

The datasets presented in this study can be found in online repositories. The names of the repository/repositories and accession number(s) can be found below: ABIDE I - https://fcon_1000.projects.nitrc.org/indi/abide/abide_I.html and ABIDE II - https://fcon_1000.projects.nitrc.org/indi/abide/abide_II.html.

## Footnotes

^1^ http://fcon_1000.projects.nitrc.org/indi/cmi_healthy_brain_network/

^2^ https://nilab-uva.github.io/AOMIC.github.io/
