# Supplementary material for: VAE deep learning model with domain adaptation, transfer learning and harmonization for diagnostic classification from multi-site neuroimaging data
Source: Front Neuroinform. 2025 Sep 11;19:1553035. doi: 10.3389/fninf.2025.1553035 (PMC12460464; doi:10.3389/fninf.2025.1553035)
Supplement: Supplementary file 1 [file Data_Sheet_1.docx]

**SUPPLEMENT**

**Effects of Combining Harmonization (ComBat) with Domain Adaptation and Transfer Learning**


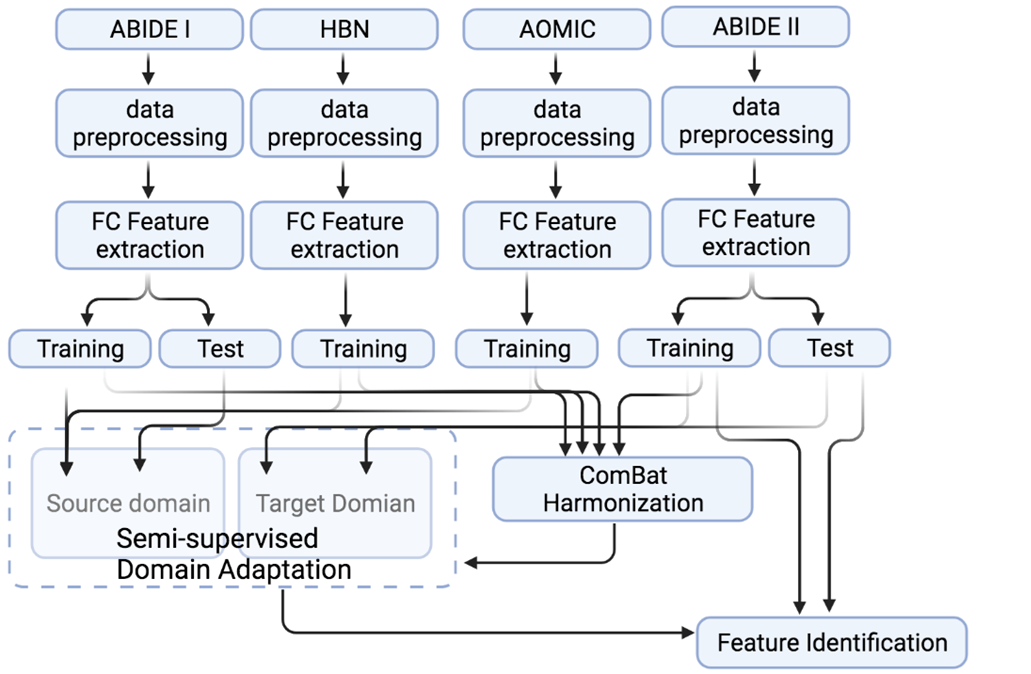


Figure S1. *Illustration of the extended model pipeline combining semi-supervised domain adaptation with ComBat harmonization.*

As shown in Fig S1, data from ABIDE I, HBN, AOMIC, and ABIDE II were preprocessed identically and used for FC feature extraction. ABIDE I served as the labeled source domain, while ABIDE II was the unlabeled target domain. HBN and AOMIC (healthy controls only) were used as additional training data to support transfer learning. Functional connectivity features from all datasets were harmonized using the ComBat method prior to training. The harmonized data were then used to train the semi-supervised domain adaptation model and perform transfer learning, allowing comparison against models trained without ComBat to assess its additional impact on classification performance.

Table S1. Classification performance of additional model combinations incorporating ComBat harmonization, MMD regularization, and TL.

| Classification Accuracy | Source Training | Source Test | Target Training | Target Test (F1-score) |
| --- | --- | --- | --- | --- |
| VAE+MMD+COMBAT | 100% | 52.53% | 83.11% | 74.6% (0.47) |
| VAE+MMD+COMBAT +TL | 98.79% | 52.53% | 82.11% | 75.4% (0.44) |

Table S1 provides additional analyses combining domain adaptation (VAE+MMD) with ComBat harmonization, both with and without transfer learning. As shown in Table S1, combining domain adaptation with ComBat harmonization alone (VAE+MMD+ComBat) achieves 74.6% accuracy in the target domain. Combining transfer learning with ComBat harmonization (VAE+MMD+ComBat+TL) further improves classification performance, increasing target domain accuracy to 75.4%. While our main analysis emphasizes that domain adaptation with transfer learning outperforms statistical harmonization alone, these supplementary results suggest that integrating ComBat with domain adaptation—either with or without transfer learning—can further enhance classification performance.


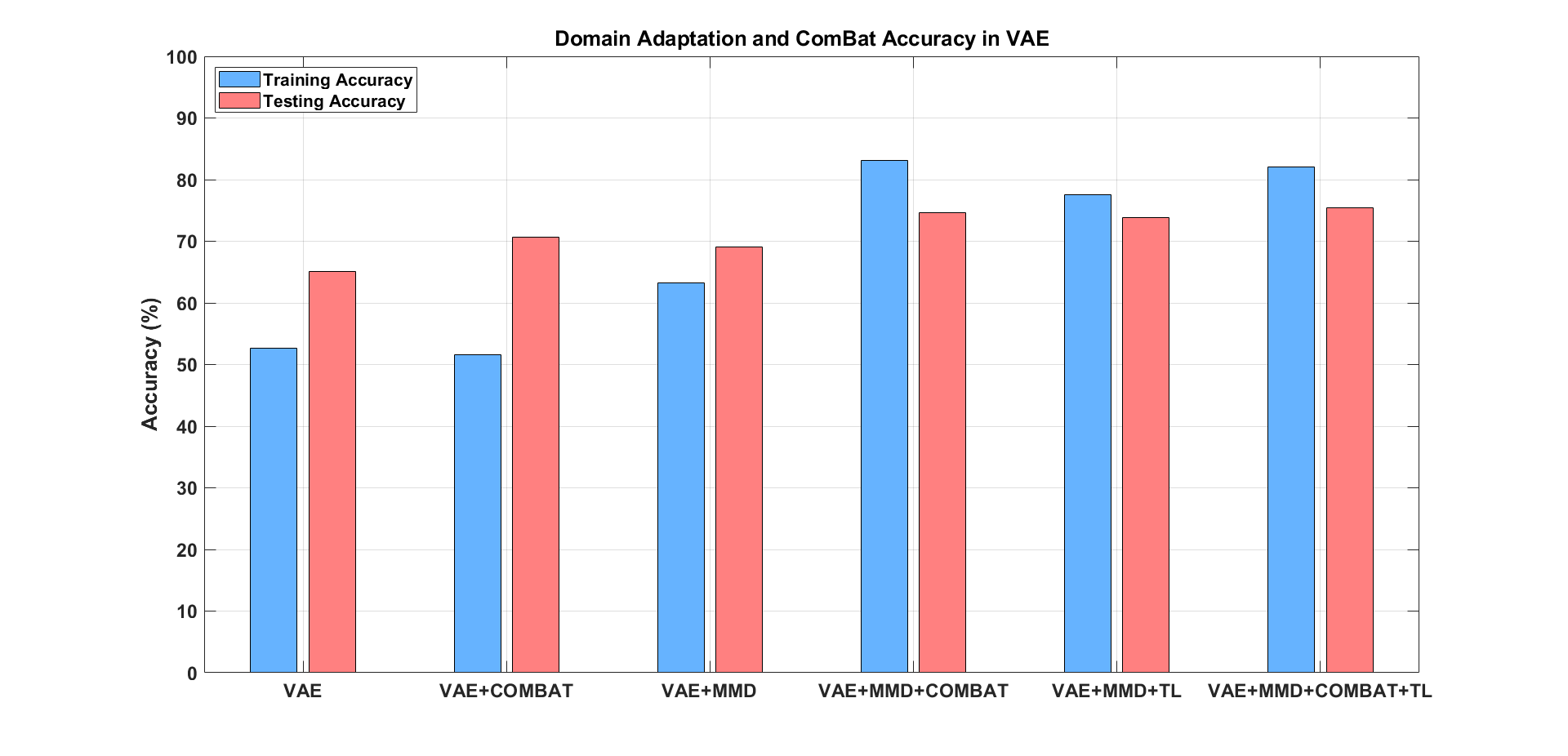


Figure S2. *The classification accuracy using different approaches combined with domain adaptation and ComBat harmonization. Blue bars refer to the training accuracy in the target domain, and red bars refer to the testing accuracy in the target domain.*

Figure S2 illustrates classification performance in the target domain across various model configurations. Adding ComBat harmonization to VAE-based models consistently improves performance, with the highest testing accuracy observed when both ComBat and transfer learning are combined with domain adaptation (VAE+MMD+ComBat+TL).

**Effect of Dataset Source in Transfer Learning within the VAE+MMD+ComBat Framework**

Table S2 . Classification results were obtained separately, including AOMIC and HBN data in the model. While the age and gender composition of HBN were comparable to ABIDE, the AOMIC cohort was older with more proportion of females (please refer to Table 1).

| Classification Accuracy | Source Training | Source Test | Target Training | Target Test (F1-score) |
| --- | --- | --- | --- | --- |
| VAE+MMD+COMBAT ＋AOMIC | 99.18% | 56.96% | 80.11% | 74.6% (0.47) |
| VAE+MMD+COMBAT ＋HBN | 97.30% | 59.49% | 82.06% | 75.4% (0.44) |

Table S2 reports the target domain classification accuracy and F1 scores when incorporating healthy control data from either HBN or AOMIC into the VAE+MMD+ComBat model. HBN provided slightly better performance than AOMIC (75.4% vs. 74.6%), likely due to its demographic similarity to the ABIDE dataset in terms of age and gender
